# Improved perioperative outcomes and early functional recovery with 3D‐printed osteotomy guide plates in ulnar shortening osteotomy: A retrospective study

**DOI:** 10.1002/jeo2.70553

**Published:** 2025-11-28

**Authors:** Zhenyu Dong, Sha Yang, Yingqi Zhao, Yidong Duan, Changqing Zheng, Lin Guo, Jingtong Lyu

**Affiliations:** ^1^ Department of Orthopeadics/Sports Medicine Center, Key Laboratory of Sports Injury Repair and Reconstruction, Southwest Hospital Third Military Medical University Chongqing China; ^2^ PLA 93968 Military Hospital Urumqi China; ^3^ Department of Clinical Laboratory Medicine, Southwest Hospital Army Medical University (Third Military Medical University) Chongqing China; ^4^ Army 953th hospital (Shigatse Branch, Xinqiao Hospital) Army Military Medical University Shigatse China

**Keywords:** 3D printing technology, osteotomy guide plates, surgical efficacy, ulnar impaction syndrome, ulnar shortening osteotomy, wrist function recovery

## Abstract

**Purpose:**

Ulnar shortening osteotomy (USO) is the primary surgical intervention for the treatment of ulnar impaction syndrome (UIS), requiring high precision. Conventional techniques face challenges in achieving optimal outcomes. This study evaluates whether 3D‐printed patient‐specific osteotomy guide plates can improve surgical precision and functional recovery.

**Methods:**

A retrospective comparative study was conducted on patients who underwent USO, divided into the USO group (*n* = 37) and the 3D‐USO group (*n* = 20). Perioperative outcomes, radiographic precision (ulnar variance and osteotomy angle), functional recovery (visual analog scale, disability of the arm, shoulder and hand, modified mayo wrist score, grip strength and range of motion) and medical cost were assessed.

**Results:**

The 3D‐USO group demonstrated significant advantages, including reduced operative time, fluoroscopy frequency and hospital stay. Radiographically, the 3D‐USO group achieved superior precision in ulnar variance correction (−0.14 vs. −3.52 mm) and osteotomy angle (3.0° vs. 6.9°). Clinically, this group exhibited significantly better early functional recovery scores and grip strength at the 6 week and 3‐month follow‐ups. No significant difference was noted between the two groups in terms of the incidence of complications or the reoperation rates.

**Conclusion:**

3D printed osteotomy guide plates improved surgical precision and early functional recovery without increasing postoperative risks. These findings corroborate the potential of the 3D‐printed osteotomy guide plate as a translational and therapeutic tool for the effective, accurate, and personalised treatment of patients with UIS.

**Level of Evidence:**

Level IV.

## Graphical abstract

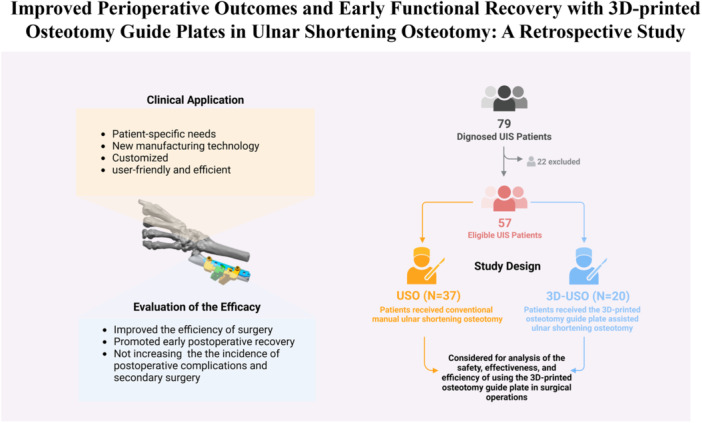

**Abbreviations**
3D‐USO: 3D‐printed osteotomy guide plate‐assisted ulnar shortening osteotomy
AWP: arthroscopic wafer procedure
CRPS: complex regional pain syndrome
CT: computed tomography
DASH: disability of the arm, shoulder and hand
DRUJ: distal radioulnar joint
IRB: Institutional Review Board
MMWS: modified mayo wrist score
MRI: magnetic resonance imaging
ROM: range of motion
STL: stereolithography
TFCC: triangular fibrocartilage complex
UG: unigraphics
UIS: ulnar impaction syndrome
USO: ulnar shortening osteotomy
VAS: visual analog scale

## INTRODUCTION

Ulnar impaction syndrome (UIS) is a common degenerative source of ulnar‐sided wrist pain, often associated with positive ulnar variance (PUV) [49, 51]. PUV increases ulnocarpal load, leading to chondral lesions and soft‐tissue injuries [22, 39, 41]. Dynamic ulnar variance may also contribute to UIS even in neutral or negative ulnar wrists [1, 41]. For refractory cases, surgical options include the arthroscopic wafer procedure or ulnar shortening osteotomy (USO) [5, 36, 44, 49, 53]. USO is widely used as first‐line treatment due to its broad applicability and technical feasibility [8, 39, 42, 53]. According to different literature data, about 73%–94% of patients are satisfied or very satisfied with the surgical results [5, 6], but it is at risk of complications, including delayed union, nonunion, hardware irritation and chronic pain [16, 44, 53].

Although technical innovations have aimed to improve accuracy [14, 18, 21, 24, 29, 37, 38, 46], conventional USO systems remain limited in achieving patient‐specific anatomical matching. Further development of individualised instrumentation may help reduce complications and improve outcomes. Three‐dimensional (3D) printing technology offers a potential solution to these challenges [4, 15, 20, 33, 40]. By utilising patient‐specific anatomical data derived from CT imaging, it enables the fabrication of individualised osteotomy guide plates [17, 34, 50]. Such patient‐specific instruments may facilitate preoperative planning of osteotomy length and orientation, and enhance intraoperative precision, potentially reducing complications associated with conventional USO and improving clinical outcomes. While previous studies have demonstrated promising results with 3D‐printed guides in various orthopaedic procedures [26, 30, 31, 34, 54], it is not clear whether the application of 3D‐printed patient‐specific osteotomy guide plates actually translates into more clinical benefits and fewer adverse events in clinical practice compared to conventional free‐hand surgical treatment.

This study aimed to retrospectively analyse eligible surgical cases to compare 12‐month outcomes between conventional USO and 3D‐printed guide‐assisted USO, evaluating the potential influence of patient‐specific guides on the procedure's safety and efficacy profile.

## MATERIALS AND METHODS

### Study design

This study received approval from our institutional review board (Approval No.: KY2025103), which granted a waiver of informed consent. A retrospective analysis was conducted using prospectively collected data from patients who underwent USO for UIS at our institution between 2015 and 2023. Of these, all cases meeting the inclusion and exclusion criteria were reviewed, with follow‐up data spanning 12 months. As all relevant data had been previously gathered during hospitalisation, surgery and routine postoperative visits, no additional written consent was required for this analysis.

**Figure 1:**
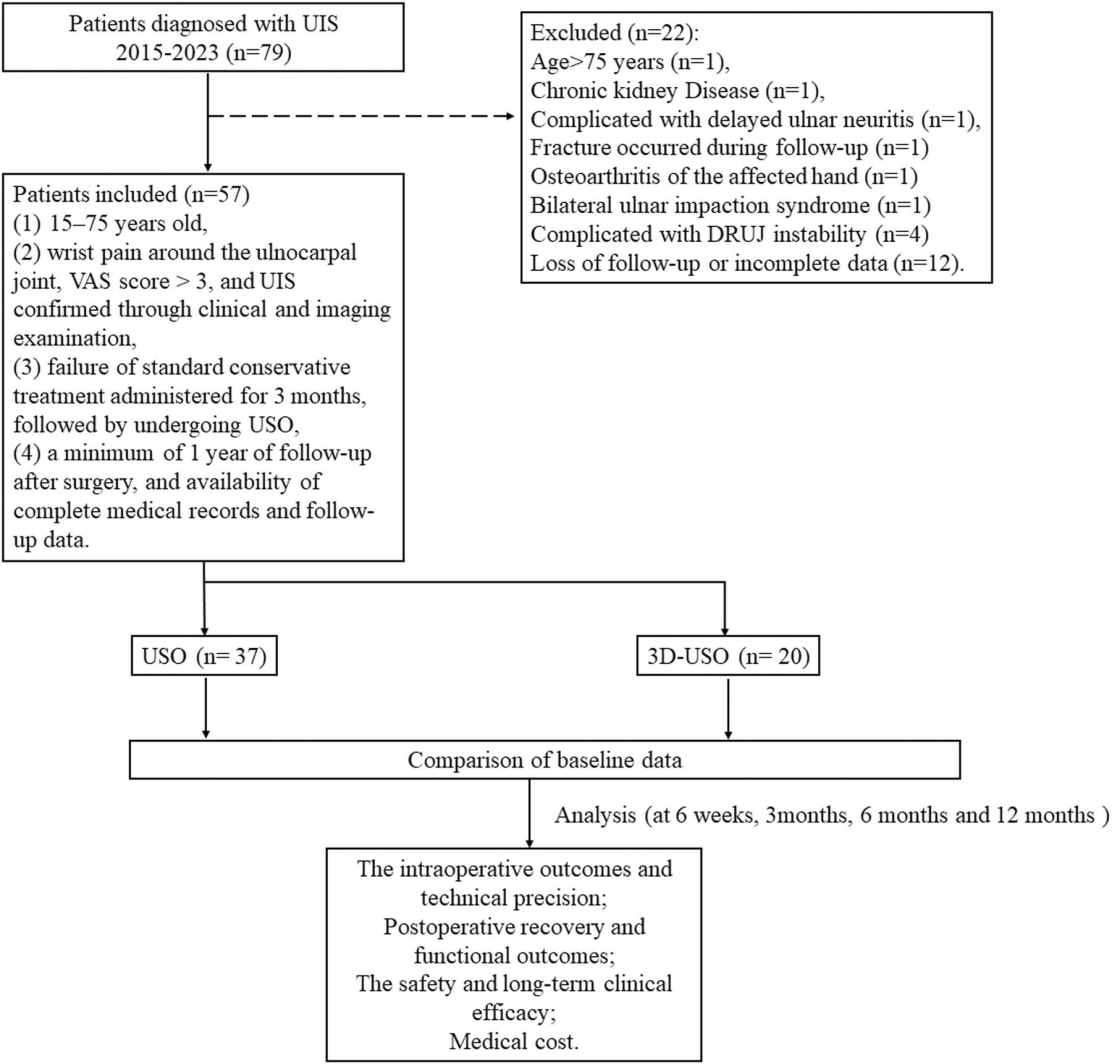
Flow chart for the procedure of study population selection and the study design. 3D‐USO, 3D‐printed osteotomy guide plate‐assisted ulnar shortening osteotomy.

The inclusion criteria are as follows: (1) patient age 15–75 years; (2) wrist pain around the ulnocarpal joint, VAS score >3, and UIS confirmed through clinical and imaging examination; (3) failure of standard conservative treatment administered for 3 months, followed by undergoing USO; (4) a minimum of 1 year of follow‐up after surgery, and availability of complete medical records and follow‐up data. The exclusion criteria were as follows: (1) the patient refused to undergo surgical treatment; (2) the presence of other malignant tumours or abnormal liver and kidney, severe blood coagulation disorders and primary cardiovascular and cerebrovascular diseases along with the concerned condition; (3) other conditions causing pain or dysfunction in the affected limb, including although not limited to fractures, osteonecrosis, acute traumatic TFCC tears, osteoarthritis, rheumatoid arthritis and cubital tunnel syndrome; (4) concomitant contralateral limb dysfunction, such as bilateral ulnocarpal impingement syndrome; (5) loss of follow‐up or refusal by the patient to participate in the study. After applying the aforementioned inclusion and exclusion criteria, a total of 57 patients were included in the final analysis (Figure 1). All patients underwent USO. The use of 3D‐printed osteotomy guides was introduced into our clinical practice in September 2020. Accordingly, among the included patients, 28 were operated on prior to this date using the freehand USO technique. After September 2020, surgical method was determined based on patient preference, resulting in 9 patients undergoing freehand USO and 20 receiving USO with 3D‐printed patient‐specific guides.

The patients were then divided into two groups: the USO group comprising 37 patients who received conventional manual osteotomy; the 3D‐USO group comprising 20 patients for whom the 3D‐printed osteotomy guide plate was used during the surgical procedure.

### Demographics and baseline variables

Consistent with previous literature, surgical outcomes and prognosis may be influenced by multiple factors. Demographic variables such as advanced age and smoking history have been identified as potential risk factors for less favourable outcomes [36]. In addition, preoperative disease severity is reasonably considered a relevant factor that may affect both efficacy and complication rates. To identify and mitigate potential confounding effects on the evaluation of surgical safety and effectiveness, the following baseline characteristics were collected and assessed: age, sex, history of hypertension, diabetes, cardiovascular disease and lifestyle factors including smoking and alcohol consumption [52]. In addition, clinical variables reflecting preoperative UIS severity—as documented in prior literature—were also collected and analysed [44]. These included symptom duration and progression, proportion of dominant hand involvement, history of trauma, PUV, grip strength as a percentage of the contralateral side [35], Palmar classification [48], range of motion, visual analogue scale (VAS) modified Mayo wrist score (MMWS) and disabilities of the arm, shoulder and hand (DASH) score [24, 28, 47]. Whether patients underwent concomitant arthroscopic wrist exploration and debridement was also recorded. All demographic information was obtained through patient self‐reporting. Smoking history was defined as consumption of at least one cigarette per day on average within the past year, and alcohol use was defined as the average daily intake of at least one alcoholic beverage. All clinical variables were assessed using previously established and widely accepted measurement methods (detailed protocols are provided in Supporting Information S1: Table S1).

### Surgical procedure and postoperative treatment

**Figure 2:**
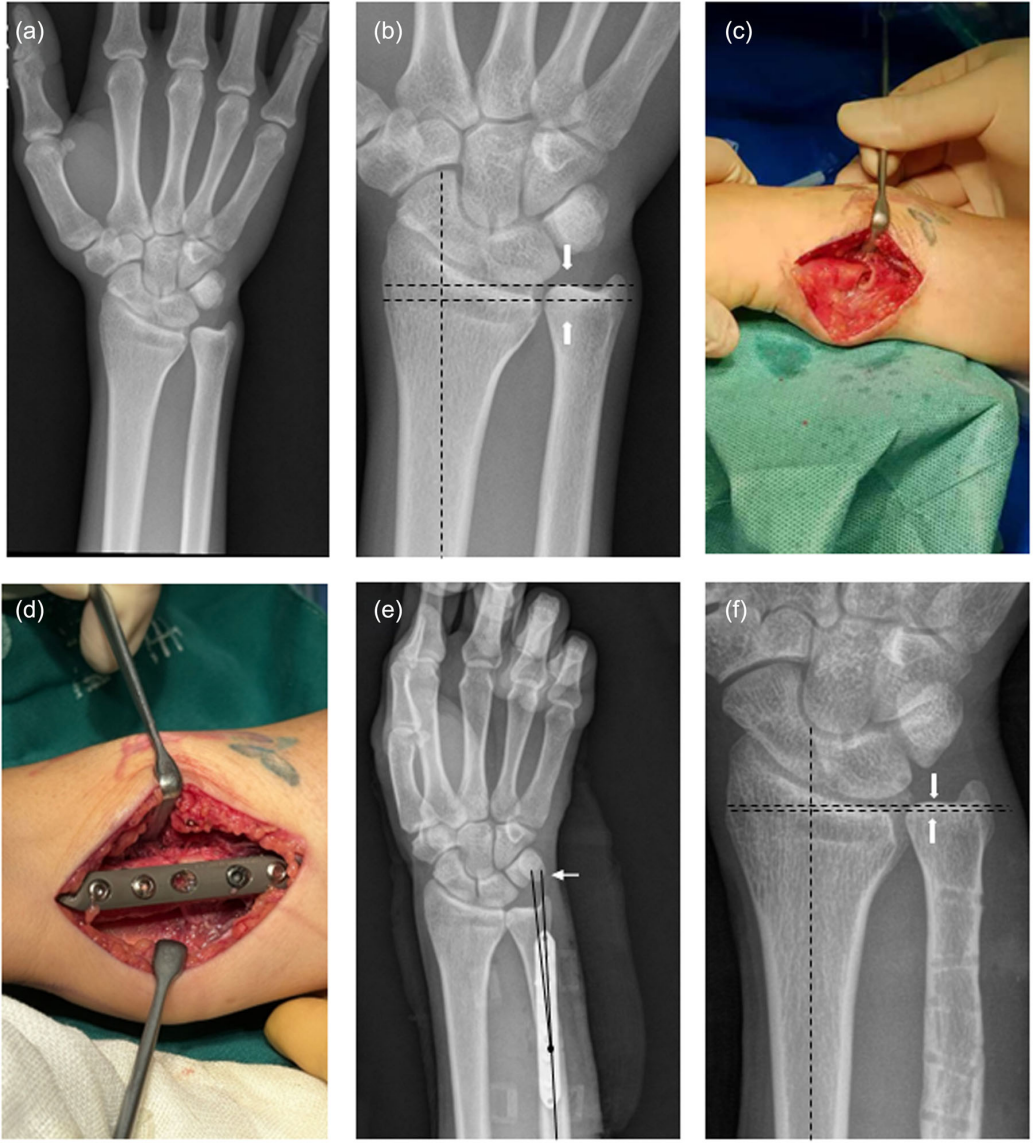
Illustration of the surgical procedure for ulnar shortening osteotomy (USO). (a) The preoperative wrist anteroposterior radiograph of a 37‐year‐old female depicting ulnar impaction syndrome in the right wrist. (b) On the radiograph, a positive ulnar variance of 3.9 mm (arrows) is visible. (c) A 4‐mm ulna osteotomy was performed to eliminate the positive ulnar variance. (d) Internal fixation using a 6‐hole reconstruction plate. (e) On the postoperative radiograph (immediately after USO), the osteotomy angle was 4.1°. (f) Satisfactory bone healing of the ulnar and a neutral variance of 0.7 mm were noted (arrows) after 14 months postoperatively.

All patients underwent USO, performed by an experienced attending surgeon with support from a standardised surgical team. For the USO group (Figure 2a–f), a 6–8 cm incision was made between the flexor and extensor carpi ulnaris tendons under general anaesthesia. A 6‐hole reconstruction plate was temporarily fixed with two cortical screws after contouring to the ulnar surface. Osteotomy was performed using an oscillating saw under saline irrigation, with the length predetermined from preoperative radiographs. After resection, compression and definitive plate fixation were achieved under fluoroscopic guidance.

**Figure 3:**
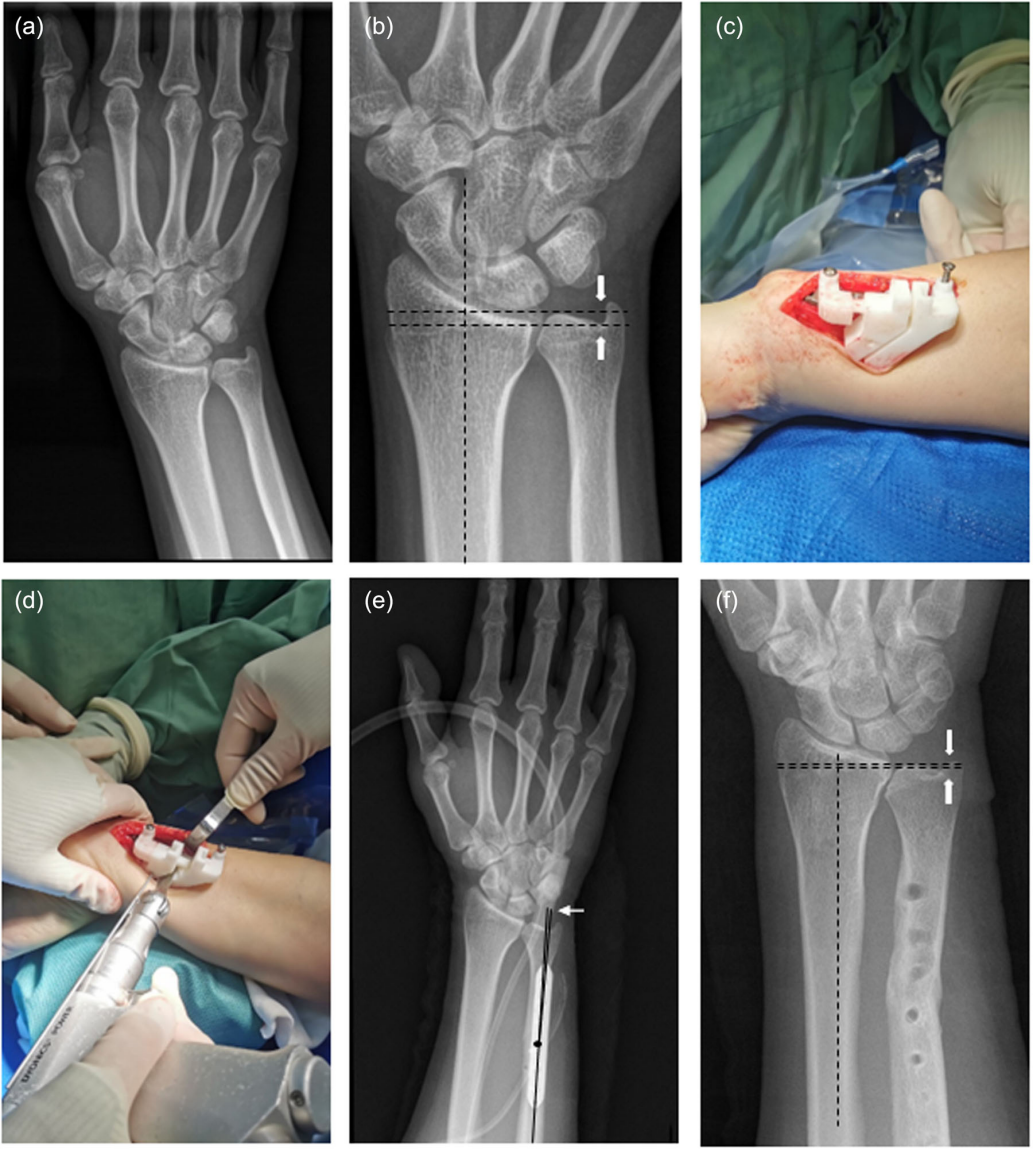
Illustration of the surgical procedure for ulnar shortening osteotomy (USO) using the 3D‐printed osteotomy plate. (a) The preoperative wrist anteroposterior radiograph of an 18‐year‐old male depicting ulnar impaction syndrome in the right wrist. (b) On the radiograph, a positive ulnar variance of 3.6 mm (arrows) was noted. (c) After determining the anatomy, the 3D osteotomy guide plate was installed. (d) A 3.6‐mm distal ulna osteotomy was performed to eliminate the positive ulnar variance. (e) On the postoperative radiograph (immediately after performing the ulnar shortening osteotomy), the osteotomy angle was 2.6° (white arrow). (f) Satisfactory bone healing of the ulnar and a neutral variance of −0.2 mm were noted (arrows) after 12 months postoperatively.

In the 3D‐USO group, patient‐specific guide plates (Figure 3a–f) were designed based on preoperative CT and manufactured via 3D printing. After exposure, the guide was fixed to the ulna using predesigned positioning pins. Osteotomy was performed through the guide slot, ensuring alignment with the planned resection length and angle.

Patients with TFCC injury (Palmer type III or higher) concurrently underwent arthroscopic debridement via standard 3–4 and 6R portals prior to osteotomy [23]. Postoperatively, all patients were immobilised with a brace for 2 weeks, followed by transition to a removable splint. Early motion of the wrist and fingers was encouraged, while load‐bearing activities were restricted. Follow‐up assessments were conducted at 6 weeks, 3, 6 and 12 months.

### Design and preparation of the 3D‐printed osteotomy guide plate

**Figure 4:**
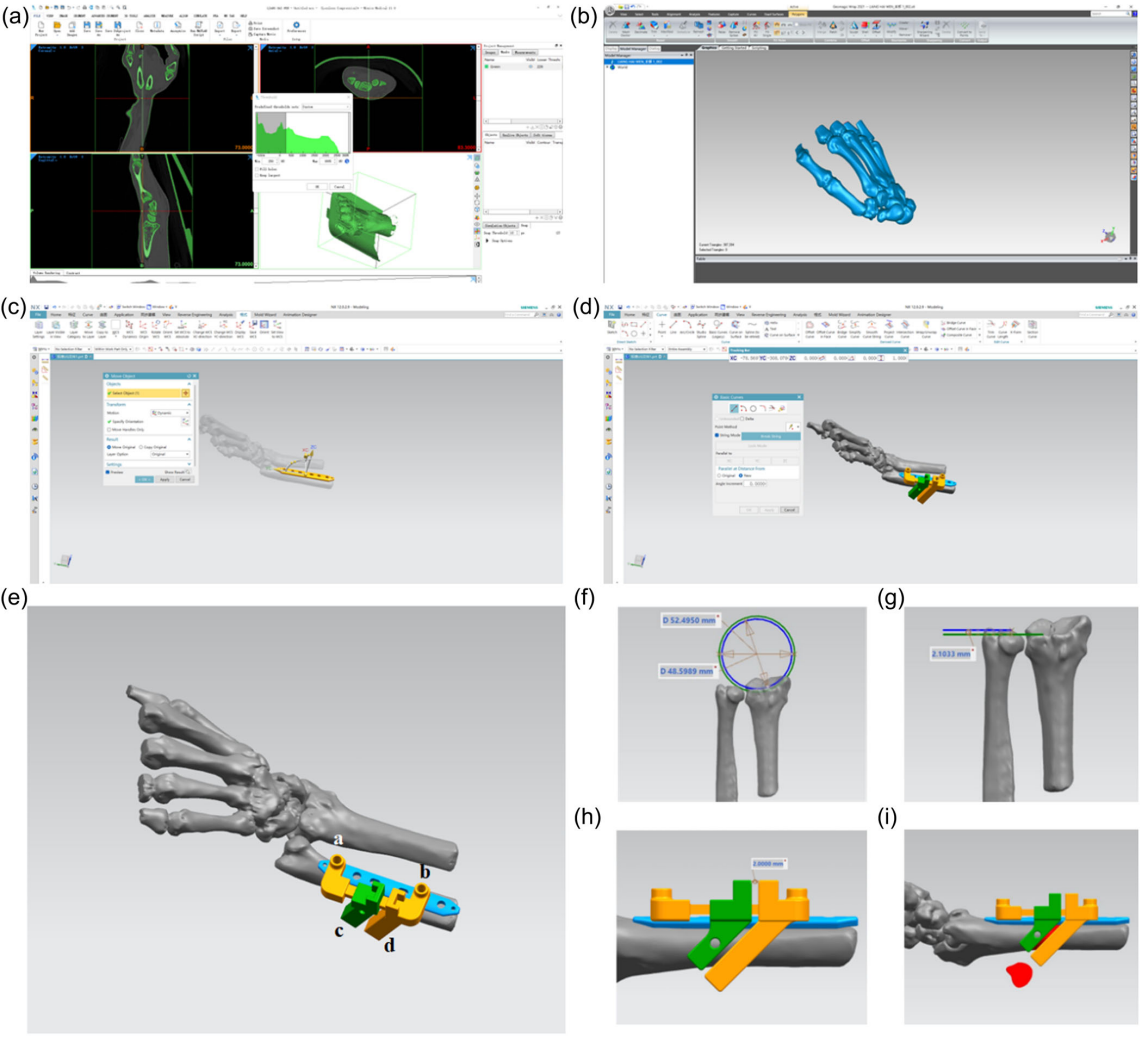
Design and manufacture of the 3D‐printed osteotomy guide plate. (a) The threshold value of 255–3005 was set for 3D wrist skeletal model reconstruction using Mimics Medical 21.0 software. (b) Surface smoothing of the 3D wrist skeletal model using Geomagic 21 software. (c) The 6‐hole plate was added to the 3D skeletal model using UG 12.0 software. (d) The 3D‐printed osteotomy guide plate matching the 3D skeletal model. (e) Details of the 3D‐printed osteotomy guide plate. The main components of the plate included (i) a distal fixed hole, (ii) a proximal fixed hole, (iii) a sliding guide block, and (iv) a fixed guide block. The ulnar model positive variation was measured. (f, g) The distance between the sliding guide block and the fixed guide block selected based on the ulnar model positive variation. (h, i) Simulated surgery to ensure the applicability of the 3D‐printed osteotomy guide plate model.

The design workflow is summarised in Figure 4a–i. Preoperative CT images (DICOM format) were segmented using a bone‐specific threshold (‘Bone (CT) 255–3005’) to reconstruct a 3D model of the ulna. After smoothing and refinement in Geomagic 21, the model was imported into UG12.0. A virtual 6‐hole plate was positioned, and a customised osteotomy guide was designed to attach to holes 1 and 5 of the plate model. The guide incorporated slots for osteotomy and drill guides, with the resection length determined based on ulnar variance. The final guide was printed after virtual validation and fitness assessment on the bone model (equipment details are provided in Supporting Information S1: Table S2).

### Outcome measures

The outcome measures and evaluation time points are shown in Supporting Information S1: Table S3. All patients were followed up at 6 weeks, 3 months, 6 months and 12 months after surgery.

The assessment of perioperative outcomes and technical precision was based on the following criteria: surgical duration, intraoperative blood loss, number of intraoperative fluoroscopies, and osteotomy accuracy evaluated from radiographs. Intraoperative blood loss was calculated using the formula: blood loss = ΔHCT × weight × 7% ÷ HCTpre [13]. Specifically, the accuracy of the osteotomy was evaluated by postoperative correction of ulnar variance and the osteotomy angle (planed vs. achieved) [27]. In the case of patients with positive ulnar variation [7], the goal was to achieve a neutral variation of 1 or –1 mm on postoperative radiographs [23]. The osteotomy angle was defined as the angle between the osteotomy end and the ulnar shaft [9]. This angle is indicative of the parallelism of the osteotomy surface after surgery. Theoretically, the osteotomy angle is close to 0°, and the osteotomy plane is nearly parallel.

Postoperative recovery and functional outcomes measures determined in the present study included the length of stay, ROM [10], grip strength [35], visual analog score (VAS), MMWS and DASH score [2, 32, 52]. The ROM and grip strength for both limbs were measured using a goniometer and a grip strength metre respectively, with the results averaged over three trials. Measure separately on the affected side and the healthy side, and use the percentage of the affected side relative to the healthy side as an objective parameter for functional recovery.

Haemorrhage, infection, iatrogenic neurovascular injury, iatrogenic tendinopathy, ulna nonunion, hardware failures (such as screw pullouts, broken screws and plate fractures), and requirement for a secondary surgery due to any reason were the variables that were recorded and analysed as complications to evaluate the safety and long‐term clinical efficacy [47].

### Bias control

Although the present comparative analysis was conducted retrospectively, the clinical and radiographic outcome data were collected prospectively under standardised protocols as part of an institutional registry for patients undergoing USO. This prospective data collection mechanism was critical for minimising assessment bias. Throughout the follow‐up period, all evaluations were performed by three independent physicians who were blinded to the specific surgical technique employed (conventional USO vs. 3D‐printed guide‐assisted USO). This was achieved by using standardised data collection forms that contained no information regarding the surgical group assignment. Each evaluator performed measurements independently according to predefined protocols. Final assessments were then reviewed by a senior attending physician to ensure consistency. This process ensured that the individuals recording the primary outcome data were unaware of the group allocations, thereby preserving the blinding integrity during data acquisition.

### Statistical analysis

The distribution of continuous variables, including age, symptom duration, ulnar variance, grip strength as a percentage of the contralateral side, ROM relative to the contralateral side, and clinical scores, was assessed for normality and homogeneity of variance using Levene's test [19, 55]. Variables with a Levene's test *p*‐value ≥ 0.05 were considered to satisfy both normality and homogeneity of variance, and were expressed as mean (SD) and compared using the independent samples *t*‐test. Those with *p* < 0.05 were considered to have heterogeneous variances, summarised as median (min, max), and compared using the Wilcoxon rank‐sum test. The categorical variables, such as certain baseline characteristics and the complications of patients, were compared between the groups using the chi‐squared test or Fisher exact test. The occurrence of any postoperative complications was considered the endpoint event in the survival analysis, which was conducted using a Log‐rank test. The differences with *p* < 0.05 were considered statistically significant. The differences with *p* < 0.01 were considered extremely significant.

A priori sample size calculation was not performed because this was a retrospective study based on a fixed cohort of patients who met the inclusion criteria during the defined study period. However, a post hoc power analysis was conducted using the observed effect size of the primary outcome measure with an alpha level of 0.05. Cohen's *d* was used as the effect size measure for the power analysis [3, 47]. Cohen's *d* values of 0.2, 0.5 and 0.8 are generally considered thresholds for small, medium and large effect sizes, respectively [12, 45]. The formula used for calculation was: Cohen's *d* = (mean₁ − mean₂)/pooled standard deviation, where the pooled standard deviation is defined as $\surd [\frac{\left(N1-1\right)*\mathrm{SD}1^{2}+\left(N2-1\right)*\mathrm{SD}2^{2}}{N1+N2-2}]$ [11]. The achieved power was calculated based on the given alpha, sample size and effect size.

Statistical analyses and data visualisation were performed using the R software (R 4.4.1) and GraphPad Prism10.2.3. The post hoc power analysis was performed using G*Power (https://www.psychologie.hhu.de/arbeitsgruppen/allgemeine-psychologie-und-arbeitspsychologie/gpower). Data preprocessing was performed using the ‘dplyr’, ‘tidyr’ and ‘data.table’ packages, while the statistical analyses were performed using the ‘stats’ and ‘survival’ packages. Visualisations were generated using the ‘ggplot2’ and ‘survminer’ packages.

### The use of artificial intelligence

During the preparation of this work, the authors used DeepSeek‐R1 (https://www.deepseek.com/) exclusively to improve linguistic clarity and grammatical accuracy. The AI was applied solely to translate and polish text, without altering or generating scientific content. All inputs were de‐identified text fragments from the authors' drafts, and every AI‐outputted suggestion was rigorously reviewed and selectively incorporated by the authors, who assume full responsibility for the final content. This use of AI adhered to academic integrity guidelines and involved no financial or conflicting interests.

## RESULTS

### Demographics and baseline data

The demographic data of the patients were presented in Table 1. No significant differences were identified in age, gender, lifestyle factors or comorbidities. Furthermore, preoperative indicators reflecting the severity of UIS, such as symptom duration, ulnar variance, grip strength, VAS score, MMWS and DASH score, also showed comparable baseline levels between the groups.

**Table 1:** Baseline characteristics of patients in the two groups.

Variable	USO (*n* = 37)	3D‐USO (*n* = 20)	*p*
Age, year	39.0 [19.0, 70.0]	35 [19.0, 66.0]	0.66
Gender			>0.99
Female	16 (43.2%)	8 (40.0%)	
Male	21 (56.8%)	12 (60.0%)	
Symptom duration, months	29.6 (8.32)	31.2 (7.76)	0.46
Duration of symptom aggravation, months	2.73 (1.30)	3.33 (1.56)	0.273
Dominant/Nondominant			>0.99
Dominant	21 (53.8%)	11 (55.0%)	
Nondominant	16 (43.2%)	9 (45.0%)	
History of trauma, months			0.59
No	18 (48.6%)	12 (60.0%)	
Yes	19 (51.4%)	8 (40.0%)	
Hypertension			>0.99
No	33 (89.2%)	18 (90.0%)	
Yes	4 (10.8%)	2 (10.0%)	
Diabetes mellitus			>0.99
No	35 (94.6%)	19 (95.0%)	
Yes	2 (5.4%)	1 (5.0%)	
Cardiopathy			0.76
No	35 (94.6%)	20 (100.0%)	
Yes	2 (5.4%)	0 (0.0%)	
Smoking			0.94
No	26 (70.3%)	15 (75.0%)	
Yes	11 (29.7%)	5 (25.0%)	
Drinking			>0.99
No	34 (91.9%)	19 (95.0%)	
Yes	3 (8.1%)	1 (5.0%)	
Preoperative ulnar variance, mm	3.98 (0.79)	3.87 (0.83)	0.86
Preoperative grip strength, %	55.4 (15.2)	58.2 (13.0)	0.48
Palmar class			0.37
2A	0 (0.0%)	0 (0.0%)	
2B	15 (40.5%)	6 (30.0%)	
2C	18 (48.6%)	12 (60.0%)	
2D	4 (10.8%)	2 (10.0%)	
2E	0 (0.0%)	0 (0.0%)	
Preoperative ROM			
Flexion, %	78.3 (10.5)	79.2 (11.2)	0.76
Extension, %	76.4 (10.8)	78 (10.5)	0.41
Pronation, %	70.6 [27.2, 91.2]	68.2 [23.9, 93.5]	0.64
Supination, %	65.3 (13.9)	67.3 (13.5)	0.60
Ulnar deviation, %	46.4 (16.2)	48.7 (15.8)	0.60
Radial deviation, %	58.8 [29.4, 94.7]	63.2 [25.1, 93.6]	0.50
Preoperative clinical assessment, points			
VAS score	6.0 [3.0, 9.0]	6.0 [2.0, 9.0]	0.97
MMWS	45 [30.0, 60.0]	49.0 [20.0, 60.0]	0.06
DASH score	45.0 (13.1)	48.2 (11.8)	0.43
Combined with arthroscopic treatment			0.31
No	30 (81.1%)	13 (65.0%)	
Yes	7 (18.9%)	7 (35.0%)	

*Note*: The continuous variables that conformed to a normal distribution were expressed as means (SD), while the variables that did not conform to a normal distribution were expressed as medians (min, max). The categorical variables were presented using count (%), and the percentages (%) were relative to the contralateral side. *p*‐Values were based on the results of the independent two sampled *t*‐test or Wilcoxon rank sum test for continuous variables and *χ* ^2^ test for categorical variables. *p* < 0.05 was considered the threshold of statistical significance.

Abbreviations: MMWS, modified mayo wrist score; 3D‐USO, 3D‐printed osteotomy guide plate‐assisted ulnar shortening osteotomy; ROM, range of motion; USO, ulnar shortening osteotomy; VAS, visual analog scale.

### Perioperative outcomes and technical precision

**Figure 5:**
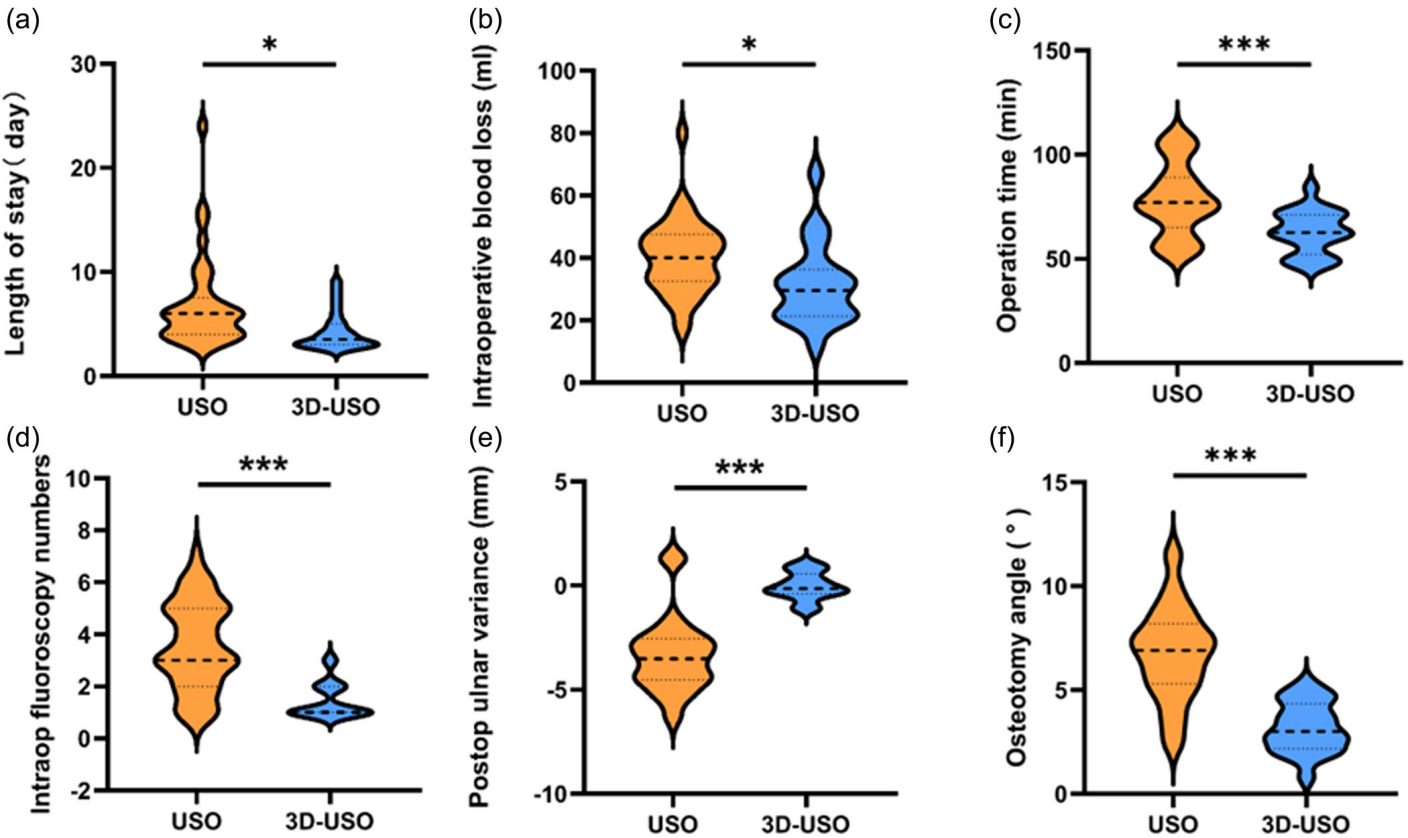
Comparison of intraoperative outcomes and technical precision between the two groups. (a–f) The distribution of surgical duration, intraoperative blood loss, length of stay, intraoperative X‐ray fluoroscopies, postoperative ulnar positive variation, and osteotomy angle, respectively. These variables did not follow a normal distribution and were, therefore, compared using the Wilcoxon rank sum test. *p < 0.05, ***p < 0.001.

The 3D‐USO group demonstrated advantages in hospital stay (3.5 [3.0, 9.0] vs. 6.0 [3.0, 24.0] days, *p* = 0.002) and intraoperative blood loss (29.5 [12.0, 67.0] vs. 40.0 [17.0, 80.0] mL, *p* = 0.015) compared to the USO group (Figure 5a,b). A notable reduction was also observed in both operative time (62.5 [47.0, 84.0] vs. 77.0 [53.0, 110] min, *p* < 0.001) and the number of intraoperative fluoroscopies (1.0 [1.0, 3.0] vs. 3.0 [1.0, 7.0], *p* < 0.001) with the application of 3D‐printed osteotomy guides (Figure 5c,d). Regarding technical precision, postoperative assessment indicated superior accuracy in the 3D‐USO group. The median UPV was −0.14 [−1.17, 1.07] mm, closely aligning wWith the surgical goal of neutral variance, in contrast to −3.52 [−6.45, −1.20] mm in the conventional group (Figure 5e). Furthermore, the median angular deviation of the osteotomy was significantly lower in the 3D‐guided group (3.0° [0.8, 5.3]°) compared to the conventional USO group (6.9° [2.4, 11.6]°, *p* < 0.001 for both comparisons; Figure 5f). These findings collectively suggest that the 3D‐printed guide technique may contribute to improved perioperative efficiency and enhanced surgical precision. Detailed statistical results are provided in Supporting Information S1: Table S4.

### Postoperative recovery and functional outcomes

**Figure 6:**
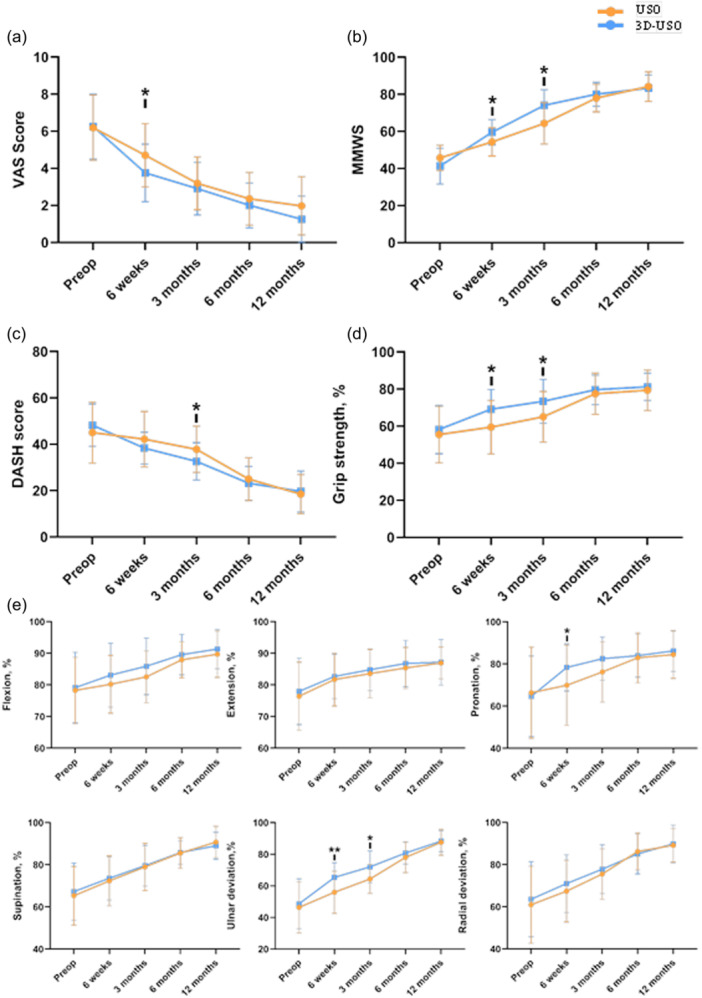
Clinical outcomes for all follow‐up periods after ulnar shortening osteotomy (USO) or 3D‐printed osteotomy guide plate‐assisted USO in patients with ulnar impaction syndrome (UIS). According to the preoperative measurements, (a) the percentage of grip strength in the healthy hand, (b) the visual analog scale score, (c) modified mayo wrist score, (d) disability of the arm, shoulder and hand score and (e) the percentage of wrist range of motion in the healthy hand, for the two groups of patients, at 6 weeks, 3 months, 6 months and 12 months postsurgery. The blue line represents the 3D‐USO group. The yellow line represents the USO group. The data presented are mean ± SE. Data were analysed using an independent sample *t*‐test for the normally distributed variables and a Wilcoxon rank‐sum test for the nonnormally distributed variables. *p < 0.05, **p < 0.01.

Postoperative recovery and functional outcomes were evaluated using patient‐reported outcomes (VAS, MMWS and DASH scores) alongside objective measures including grip strength and ROM, each expressed as a percentage of the contralateral healthy side. At the 6‐week follow‐up, the 3D‐USO group showed a lower VAS score (3.5 [1.0, 7.0]) compared to the USO group (5.0 [1.0, 7.0], *p* = 0.030). MMWS scores were also higher in the 3D‐USO group at both 6 weeks (60.0 [50.0, 70.0] vs. 55.0 [40.0, 70.0], *p* = 0.022) and 3 months (75.0 [55.0, 90.0] vs. 65.0 [45.0, 85.0], *p* = 0.002). Similarly, the DASH score was lower in the 3D‐USO group at 3 months (32.8 ± 8.07 vs. 37.8 ± 10.0, *p* = 0.037), suggesting better patient‐perceived function. Figure 6a–c illustrates the differences in VAS, MMWS and DASH scores between the two groups. Objectively, grip strength recovery (Figure 6d) was greater in the 3D‐USO group at 6 weeks (69.2 ± 10.7% vs. 59.4 ± 14.4%, *p* = 0.006) and 3 months (73.4 ± 11.8% vs. 65.1 ± 13.7%, *p* = 0.05). Regarding ROM (Figure 6e), the 3D‐USO group exhibited improved pronation (80.0 [55.0, 97.3]% vs. 75.3 [33.2, 96.1]%, *p* = 0.0497) and ulnar deviation (64.3 [51.3, 86.3]% vs. 58.9 [31.3, 85.3]%, *p* = 0.008) at 6 weeks, with the advantage in ulnar deviation persisting at 3 months (72.0 ± 10.1% vs. 64.3 ± 9.1%, *p* = 0.007). There were no significant differences in the movement directions of other joints between the two groups. These findings collectively indicate that the use of 3D‐printed guides may contribute to enhanced functional recovery in the early term postoperative phases. Detailed statistical data are provided in Supporting Information S1: Table S5.

### Safety and long‐term clinical efficacy

**Figure 7:**
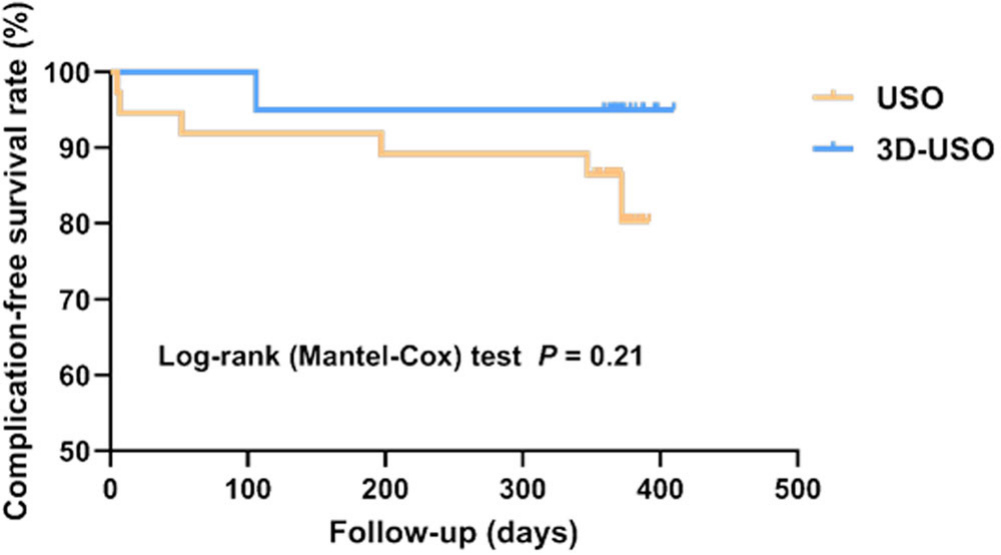
Complications‐free survival curves for the two groups. The blue line represents the 3D‐printed osteotomy guide plate‐assisted ulnar shortening osteotomy (3D‐USO) group. The yellow line represents the USO group. Differences were evaluated based on the Log‐rank test statistics.

As visible in Table 2, no significant differences were noted between the two groups in terms of the incidence of complications or the reoperation rates (*p*_Complications_ > 0.99, *p*_Secondary surgery_ = 0.52). Meanwhile, a Kaplan–Meier survival analysis was conducted for the two groups, using procedural complications and time‐to‐event as endpoints (Figure 7), which revealed similar results (*P*_log‐rank test_ = 0.21). Overall, these results indicated no difference in procedural complications between the two groups.

**Table 2:** Comparison of surgical complications between the two groups.

Complications	USO (*n* = 37)	3D‐USO (*n* = 20)	*p*
Infection	1 (2.7%)	0 (0.0%)	>0.99
Iatrogenic vascular injuries	1 (2.7%)	0 (0.0%)	>0.99
Iatrogenic nerve injuries	1 (2.7%)	0 (0.0%)	>0.99
Iatrogenic tendinopathy	1 (2.7%)	0 (0.0%)	>0.99
Ulnar nonunion	1 (2.7%)	0 (0.0%)	>0.99
Hardware failures	1 (2.7%)	1 (5.0%)	>0.99
Total complications (*N*, %)	6 (16.2%)	1 (5.0%)	>0.99
Unscheduled secondary surgery (*N*, %)^a^	3 (8.1%)	0 (0.0%)	0.52

*Note*: *p* values are from the Fisher test.

Abbreviations: 3D‐USO, 3D‐printed osteotomy guide plate‐assisted ulnar shortening osteotomy; USO, ulnar shortening osteotomy.

^a^ A total of three cases underwent secondary surgery. In two of these patients, secondary surgery was necessary due to the nonunion of the osteotomy. Another patient complained of postoperative pain due to residual positive ulna variation and had to undergo a second surgery.

## DISCUSSION

In this study, a novel, convenient and customised 3D‐printed osteotomy guide plate was developed, and its efficacy was also evaluated. It was revealed that the use of this customised template may be associated with several significant improvements in several key areas.

The 3D‐USO group demonstrated advantages in hospital stay and intraoperative blood loss, which may reflect reduced iatrogenic tissue damage attributable to the patient‐specific design derived from preoperative imaging. Additionally, operative time and fluoroscopy frequency were notably lower in the 3D‐USO group, suggesting that preoperative planning based on individual anatomy could simplify the surgical procedure and minimise radiation exposure. More importantly, the 3D‐printed osteotomy template contributed to significantly improved accuracy in achieving neutral ulnar variance and optimal osteotomy alignment—key objectives of USO. These observations highlight the potential of 3D‐printed osteotomy template to enhance surgical reproducibility and reduce variability associated with freehand techniques, possibly leading to more consistent and predictable corrections.

Over the 1‐year follow‐up period, observations suggested a trend towards earlier functional recovery in the 3D‐USO group compared to the USO group. Patient‐reported outcomes at 6 weeks and 3 months postoperatively appeared more favourable in the 3D‐USO group, as reflected by lower VAS and DASH scores, alongside consistently higher MMWS scores at both time points. These subjective reports were supported by objective functional measurements. Specifically, grip strength recovery was significantly better in the 3D‐USO group at 6 weeks, and improvements in ROM—particularly in ulnar deviation and pronation—were also observed at the 6‐week and 3‐month assessments. The possible reason for this could be the higher accuracy of osteotomy and fixation in the 3D‐USO group compared to the traditional USO group, which led to reduced damage to the surrounding tissues, mitigated postoperative inflammatory response and swelling, and improved recovery of muscle function. Interestingly, over time, the differences in the above indicators between the groups were eliminated. As healing progresses, the influence of the inherent biological repair process and confounding variables—such as rehabilitation adherence and individual patient factors—increases. This may explain the gradual attenuation of differences between the groups over time, potentially masking the initial advantage conferred by the surgical technique. The accelerated recovery in forearm pronation and wrist ulnar deviation observed in the 3D‐USO group may be attributed to the specific pathophysiology of dynamic UIS (DUIS) [25]. DUIS frequently causes pain during loaded pronation and ulnar deviation—movements that narrow the ulnocarpal space [1]. The precise osteotomy enabled by the 3D‐printed guide likely restored more physiological ulnocarpal alignment, thereby reducing mechanical impingement and pain during these functionally critical motions. This biomechanical improvement may have encouraged earlier and more confident mobilisation specifically in these previously pain‐provoking directions. The absence of similarly pronounced differences in other movement planes suggests that the benefit of patient‐specific guidance is most evident in actions most affected by ulnar impaction. Collectively, these results point to improved early functional recovery with the use of patient‐specific 3D‐printed guides in USO.

Moreover, the only complication observed in the 3D‐USO group occurred in a 29‐year‐old military man, who presented with screw loosening and fracture and skin surface protrusion. The reason for this could be the occupation of this patient, which rendered it necessary to begin heavy military training as soon as possible, which triggered the complications. No significant differences were observed between the two groups in terms of complication or reoperation rates. This finding may be attributed primarily to two considerations. First, the present study may have been underpowered to detect differences in these relatively low‐frequency events. Complications such as nonunion or hardware irritation occurred in only a small number of cases, and a larger sample size would be required to conclusively demonstrate any potential reduction in their incidence. Second, conventional USO performed by experienced surgeons has already established a high standard of safety. Thus, the principal advantage of 3D‐printed guides in this context may lie not in preventing major adverse events, but rather in enhancing procedural determinacy—such as improving preoperative planning efficiency, osteotomy accuracy and facilitating early functional recovery. This distinction underscores that the value of patient‐specific technology may stem from refining efficiency and functional outcomes, rather than fundamentally altering the safety profile of an already mature surgical procedure.

To address limitations inherent in the retrospective sample size, a post hoc power analysis was performed. The results, detailed in Supporting Information S1: Tables S4 and S5, showed that while power varied, the effect sizes (Cohen's *d*) for all positive outcomes surpassed 0.5, indicating at least a medium effect. The lowest values were observed for the 3‐month DASH score (*d* = 0.555, power = 0.502) and 6‐week pronation (*d* = 0.566, power = 0.517). This overall pattern provides reassurance that the key findings are robust and not merely type I errors. Nevertheless, we acknowledge that post hoc power is derivative of the observed data, and thus these results warrant confirmation through future prospective studies with prespecified sample size calculations [43].

The findings of the present study have certain practical and clinical implications. The study demonstrated that the use of 3D‐printed osteotomy guides significantly improved surgical precision and simplified the procedure, while maintaining a safety profile comparable to that of conventional USO. The customised nature of the 3D‐printed guide plate, which is tailored to the specific anatomical structure of the patient, enabled precise surgical guidance, minimised damage to surrounding soft tissues, and facilitated a further accurate osteotomy procedure, all of which led to improved early functional recovery. Further, the guided approach allowed for a faster operation, reducing procedure duration and eliminating reliance on intraoperative radiation while enhancing the confidence and expertise of the surgeon.

However, despite promising results, the present study also has certain limitations. For instance, although we conducted a post hoc power analysis, the small sample size of the study might affect the generalisability of the results, and a study with a larger sample size is needed to generate more reliable data. In addition, studies with a longer follow‐up period have to be conducted to comprehend long‐term outcomes and potential late complications. Single‐centre studies, such as the present one, could introduce bias. Therefore, multi‐centre studies are warranted to validate outcomes across different settings and populations. Moreover, while the use of the 3D‐printed osteotomy guide plate is relatively simple, it nonetheless requires professional training, and the learning curve might influence patient outcomes. Further, our results reveal a potential phenomenon: people with relatively higher socioeconomic status are more inclined to promote emerging technologies that facilitate early recovery, even though such technologies may incur higher costs. However, cost considerations remain a crucial basis for clinical decision‐making. The cost brought by 3D‐printed osteotomy guide plate still constitutes a major constraint for their wide application at this stage. Studies exploring the above concerns while considering the patient‐specific factors would facilitate the identification of the populations that would particularly benefit from the procedure.

## CONCLUSION

The study demonstrated that using a 3D‐printed osteotomy guide plate in USO reduces surgical duration, intraoperative blood loss, fluoroscopy usage and length of hospital stay. The guide‐plate assisted surgery resulted in an ulnar variance closer to 0 mm and smaller osteotomy angles compared to free‐hand osteotomy. The guide plate also enhances wrist grip strength, lowers VAS scores and improves early postoperative functional scores. In addition, it facilitates earlier recovery of ulnar deviation and pronation. No significant differences in complication rates or unplanned secondary procedures were noted between groups. These findings suggest that 3D‐printed guide plate‐assisted USO is superior to USO alone and merits wider adoption.

## AUTHOR CONTRIBUTIONS

**Zhenyu Dong**: Investigation; methodology; software; writing—original draft. **Sha Yang**: Visualisation; writing—original draft; writing—review and editing. **Yingqi Zhao**: Formal analysis; writing—original draft. **Yidong Duan**: Investigation. **Changqing Zheng**: Investigation. **Lin Guo**: Funding acquisition; supervision; validation; data curation. **Jingtong Lyu**: Conceptualisation; data curation; resources; supervision; writing—review and editing. Lin Guo and Jingtong Lyu were jointly responsible for the overall planning and scientific integrity of the research project.

## CONFLICT OF INTEREST STATEMENT

The authors declare no conflicts of interest.

## ETHICS STATEMENT

The study was approved by the institutional ethics and review board of the First Affiliated Hospital of Army Medical University (approval no. KY2025103), and the requirement for informed consent from the patients was waived by the board. The primary justifications for waiving the requirement of informed consent are as follows: this study was conducted retrospectively, with the main data derived from historical medical records. The analysis solely involved desensitised and anonymized medical information, ensuring that no additional physical, psychological, or social harm or risk was imposed on patients throughout the entire research process.

## Supporting information

Supporting information.

## Data Availability

The data that support the findings of this study are available from the corresponding author upon reasonable request.
