# Supplementary material for: Improved perioperative outcomes and early functional recovery with 3D‐printed osteotomy guide plates in ulnar shortening osteotomy: A retrospective study
Source: J Exp Orthop. 2025 Nov 28;12(4):e70553. doi: 10.1002/jeo2.70553 (PMC12661214; doi:10.1002/jeo2.70553)
Supplement: Supplementary file 1 — Supporting information. [file JEO2-12-e70553-s001.docx]

**Table S1.** The measurement methods for the evaluation indicators of this study

| **Indicators** | **Measurement methods** | **Reference (PMID)** |
| --- | --- | --- |
| Length of stay | Based on the patient's hospital medical records calculation | None |
| Blood loss | During the operation, a tourniquet was used. However, it was impossible to accurately assess the slow leakage of blood within the surgical area after the operation. Therefore, a formula was used for the assessment. estimation method: blood loss= ΔHCT×weight×7%÷HCTpre | 22145995 |
| Fluoroscopy number | Based on the patient's hospital medical records calculation | None |
| Surgical time | Based on the patient's hospital medical records calculation | None |
| Ulnar variance | Using radiographs, we determin the osteotomy angle was the angle between the osteotomy end and the ulnar shaft to measure the parallelism of the osteotomy surface after operation.ed ulnar variance by drawing vertical lines independently along the longitudinal axis of the radius. These lines intersected at the nearest point of the distal radius's articular surface and the farthest point of the distal ulna's articular surface. The distance between these points represents ulnar variance. For patients with positive ulnar variation, the goal was to achieve a neutral variation of 1 or -1mm on postoperative radiographs. | 11796902 |
| Osteotomy angle | The osteotomy angle was the angle between the osteotomy end and the ulnar shaft to measure the parallelism of the osteotomy surface after operation. Theoretically, the osteotomy angle is close to 0°, and the osteotomy plane is nearly parallel. | 38819766 |
| ROM | The ROM and grip strength for both limbs was measured using a goniometer and a grip strength meter respectively, with the results averaged over three trials. Measure separately on the affected side and the healthy side, and use the percentage of the affected side relative to the healthy side as an objective parameter for functional recovery. The range of motion for wrist flexion, extension, ulnar deviation, and radial deviation is typically measured using a goniometer with the patient seated and the forearm stabilized. For flexion (palmar flexion) and extension (dorsiflexion), the goniometer's axis is placed on the lateral wrist near the triquetrum bone, with the stationary arm aligned parallel to the ulna and the moving arm parallel to the fifth metacarpal. The patient then moves the palm upward toward the ceiling for extension or downward toward the floor for flexion. Similarly, for ulnar and radial deviation, the axis is placed dorsally over the capitate bone. The stationary arm is aligned with the dorsal midline of the forearm, and the moving arm with the third metacarpal, as the patient moves the hand toward the little finger (ulnar deviation) or the thumb (radial deviation).  Pronation and supination, which are motions of the forearm that critically influence hand positioning, are measured with the elbow flexed to 90° and held against the body to isolate the movement. The goniometer's axis is placed near the ulnar styloid for pronation or the radial styloid for supination, with the stationary arm held perpendicular to the floor. The moving arm is aligned with the distal forearm or a held object as the patient rotates the forearm to face the palm down (pronation) or up (supination). Grip strength, a key indicator of overall hand function, is objectively measured using a hand dynamometer held with the elbow flexed at 90° and the wrist in a neutral position, with the average of 3 trials recorded. | 35753828 |
| Grip strength |  |  |

**Table S2.** Main instruments used in the designing and manufacturing of the 3D-printed osteotomy guide plate.

| **Type of instruments** | **The technical details** | **Source instrument** |
| --- | --- | --- |
| **Mimics 21.0** | For processing images and creating 3D models. | Materialise, Leuven, Belgium |
| **Geomagic 21** | For trimming and smoothing the 3D models. | Geomagic, Morrisville, USA |
| **64-row 128-slice volume**  **CT (VCT)** | For obtaining high-quality CT images with slice thickness of 1.0mm. | Philips, Amsterdam, The Netherlands |
| **UG12.0** | For model establishment, test and evaluation of ulna osteotomy guide plate, and generating efficient CNC machine tool programs to ensure the accuracy and reliability of the manufacturing process of 3D printed osteotomy guide plate. | Siemens PLM Software, Plano, USA |

Table S3. Outcome measures and evaluation time points

| **Time Point** | **Baseline** | **Surgery phase** | **Follow-up phase** | | | |
| --- | --- | --- | --- | --- | --- | --- |
|  | -1^st^ Week | 1^st _^ 21^th^ Day | 6^th^ Week | 3^rd^ month | 6^th^ month | 12^th^ month |
| Medical history | √ |  |  |  |  |  |
| Inclusion/exclusion criteria | √ |  |  |  |  |  |
| Sign informed consent | √ |  |  |  |  |  |
| **Intraoperative outcomes and technical precision measures** | | | | | | |
| Surgical duration |  | √ |  |  |  |  |
| Intraoperative blood loss |  | √ |  |  |  |  |
| Intraoperative fluoroscopy number |  | √ |  |  |  |  |
| Surgical time |  | √ |  |  |  |  |
| Ulnar variance | √ | √ |  |  |  | √ |
| Osteotomy angle |  | √ |  |  |  | √ |
| Length of stay |  | √ |  |  |  |  |
| **Postoperative recovery and functional outcome measures** | | | | | | |
| ROM | √ |  | √ | √ | √ | √ |
| Grip strength | √ |  | √ | √ | √ | √ |
| VAS | √ |  | √ | √ | √ | √ |
| MMWS | √ |  | √ | √ | √ | √ |
| DASH score | √ |  | √ | √ | √ | √ |
| **Safety and long-term clinical efficacy measures** | | | | | | |
| Complications |  | √ | √ | √ | √ | √ |
| Revision rate |  | √ | √ | √ | √ | √ |
| **Direct medical cost measures** | | | | | | |
| Preoperative imaging | √ |  |  |  |  |  |
| 3D printing facility expenses |  | √ |  |  |  |  |
| Average hospitalization expenses |  | √ |  |  |  |  |
| **Indirect medical cost measures** | | | | | | |
| Cost of postoperative complications |  |  |  |  |  | √ |
| Occupational impact |  |  |  |  |  | √ |

NOTE: The “√” indicates implementation. ROM, range of motion; VAS, visual analog score; MMWS, modified Mayo wrist score; DASH, disability of the arm, shoulder, and hand.

Table S4. Detailed statistical results about perioperative indicators.

|  | **USO (N=37)** | **3D-USO (N=20)** | **Statistic** | **P** | **Cohen's d** | **Power** |
| --- | --- | --- | --- | --- | --- | --- |
| **Length of stay (day)** |  |  |  |  |  |  |
| Median [Min, Max] | 6.0 [3.0, 24.0] | 3.5 [3.0, 9.0] | W=552 | 0.00203 | 0.698 | 0.695 |
| **Blood loss (ml)** |  |  |  |  |  |  |
| Median [Min, Max] | 40.0 [17.0, 80.0] | 29.5 [12.0, 67.0] | W=516 | 0.0152 | 0.661 | 0.648 |
| **Operation time (min)** |  |  |  |  |  |  |
| Median [Min, Max] | 77.0 [53.0, 110] | 62.5 [47.0, 84.0] | W=574 | <0.001 | 1.02 | 0.951 |
| **Fluoroscopy times** |  |  |  |  |  |  |
| Median [Min, Max] | 3.0 [1.0, 7.0] | 1.0 [1.0, 3.0] | W=640 | <0.001 | 1.495 | 0.999 |
| **Postoperative UPV (mm)** |  |  |  |  |  |  |
| Median [Min, Max] | -3.52 [-6.45, -1.20] | -0.14 [-1.17, 1.07] | W=688 | <0.001 | 3.184 | ~1.00 |
| **Osteotomy angle (°)** |  |  |  |  |  |  |
| Median [Min, Max] | 6.9 [2.4, 11.6] | 3.0 [0.8, 5.3] | W=684 | <0.001 | 1.87 | 0.999 |

**NOTE:** All the intraoperative outcome indicators were continuous variables. Before comparing the two groups, Levene's test was conducted on each of them, and it was found that the variances were not homogeneous. Therefore, the Wilcoxon rank-sum test was used for the comparison between the two groups. Cohen's d was used as the effect size index for the power analysis. The calculation formula is: Cohen's d = (Mean₁ - Mean₂) / Pooled Standard Deviation. Pooled Standard Deviation=$\surd[\frac{\left( N1-1 \right)*SD1^{2}+\left( N2-1 \right)*SD2^{2}}{N1+N2-2})]$.

**Table S5.** Detailed statistical results about outcomes of follow-ups.

|  | **USO (N=37)** | **3D-USO (N=20)** | **Statistic** | **P** | **Cohen's d** | **Power** |  |
| --- | --- | --- | --- | --- | --- | --- | --- |
| **VAS** |  |  |  |  |  |  |  |
| 6 weeks | 5.0 [1.0, 7.0] | 3.5 [1.0, 7.0] | W=498 | 0.0298 | 0.624 | 0.598 |  |
| 3 months | 3.0 [0.0, 6.0] | 3.0 [0.0, 5.0] | W=408 | 0.522 | - | - |  |
| 6 months | 2.0 [0.0, 5.0] | 2.0 [0.0, 4.0] | W=416 | 0.44 | - | - |  |
| 12 months | 2.0 [0.0,6.0] | 2.0 [0.0, 4.0] | W=469 | 0.44 | - | - |  |
| **MMWS** |  |  |  |  |  |  |  |
| 6 weeks | 55.0 [40.0, 70.0] | 60.0 [50.0, 70.0] | W=235 | 0.0222 | 0.704 | 0.703 |  |
| 3 months | 65.0 [45.0, 85.0] | 75.0 [55.0, 90.0] | W=188 | 0.00212 | 0.944 | 0.916 |  |
| 6 months | 80.0 [55.0, 90.0] | 80.0 [65.0, 95.0] | W=320 | 0.393 | - | - |  |
| 12 months | 85.0 [50.0, 95.0] | 85.0 [60.0, 95.0] | W=416 | 0.422 | - | - |  |
| **DASH score** | |  |  |  |  |  |  |
| 6 weeks | | 42.2 (11.9) | 38.3 (6.84) | t=1.55 | 0.126 | - | - |
| 3 months | | 37.8 (10.0) | 32.(8.07) | t=2.15 | 0.0371 | 0.555 | 0.502 |
| 6 months | | 27.0 [8.0, 43.0] | 22.0 [12.0, 40.0] | W=415 | 0.456 | - | - |
| 12 months | | 17.0 [7.0, 48.0] | 18.5 [7.0, 42.0] | W=342 | 0.639 | - | - |
| **Grip strength (%)** |  |  |  |  |  |  |  |
| 6 weeks | 59.4 (14.4) | 69.2 (10.7) | t<0.001 | 0.00572 | 0.741 | 0.745 |  |
| 3 months | 65.1 (13.7) | 73.4 (11.8) | t<0.001 | 0.02 | 0.635 | 0.613 |  |
| 6 months | 79.6 [41.9, 92.9] | 79.3 [66.7, 90.8] | W=337 | 0.59 | - | - |  |
| 12 months | 82.9 [44.9, 92.3] | 80.5 [64.9, 91.7] | W=354 | 0.789 | - | - |  |
| **ROM (%)** |  |  |  |  |  |  |  |
| Pronation, 6 weeks | 75.3 [33.2, 96.1] | 80.0 [55.0, 97.3] | W=288 | 0.0497 | 0.566 | 0.517 |  |
| Pronation, 3 months | 82.3 [48.4, 96.1] | 82.7 [60.3, 98.4] | W=292 | 0.197 | - | - |  |
| Pronation, 6 months | 86.8 [58.1, 98.6] | 85.0 [61.3, 99.0] | W=365 | 0.941 | - | - |  |
| Pronation, 12 months | 97.8 [59.1, 99.6] | 88.8 [62.3, 100.0] | W=348 | 0.722 | - | - |  |
| Ulnar deviation, 6 weeks | 58.9 [31.3, 85.3] | 64.3 [51.3, 86.3] | W=212 | 0.00845 | 0.627 | 0.602 |  |
| Ulnar deviation, 3 months | 64.3 (9.1) | 72.0 (10.1) | t=<0.001 | 0.00725 | 0.812 | 0.820 |  |
| Ulnar deviation, 6 months | 77.2 [50.3, 95.5] | 79.6 [66.5, 95.5] | W=310 | 0.323 | - | - |  |
| Ulnar deviation, 12 months | 89.1 [53.9, 99.9] | 88.4 [78.4, 98.7] | W=376 | 0.927 | - | - |  |

**NOTE:** All the follow-up indicators were continuous variables. Before comparing the two groups, the homogeneity of variance was evaluated by the Leven test. The distribution was represented by the mean (SD) or the median [Min, Max], and statistical analysis was conducted using the independent sample t-test or the Wilcoxon rank-sum test. Cohen's d was used as the effect size index for the power analysis. VAS: visual analogue scale; MMWS: Mayo wrist score; DASH score: disability of the arm, shoulder, and hand score; ROM: range of motion.
